# Multimorbidity and COVID-19 Outcomes in the Emergency Department: Is the Association Mediated by the Severity of the Condition at Admission?

**DOI:** 10.3390/jcm13237182

**Published:** 2024-11-26

**Authors:** Alberto Catalano, Carlotta Sacerdote, Marco Alvich, Alessandra Macciotta, Lorenzo Milani, Cinzia Destefanis, Kibrom Teklay Gebru, Barbara Sodano, Lisa Padroni, Maria Teresa Giraudo, Giovannino Ciccone, Eva Pagano, Adriana Boccuzzi, Valeria Caramello, Fulvio Ricceri

**Affiliations:** 1 Centre for Biostatistics, Epidemiology, and Public Health, Department of Clinical and Biological Sciences, University of Turin, Orbassano, 10043 Turin, Italy; alessandra.macciotta@unito.it (A.M.); lorenzo.milani@unito.it (L.M.); cinzia.destefanis@unito.it (C.D.); kibromteklay.gebru@unito.it (K.T.G.); barbara.sodano@unito.it (B.S.); lisa.padroni@unito.it (L.P.); mariateresa.giraudo@unito.it (M.T.G.); fulvio.ricceri@unito.it (F.R.); 2 Department of Translational Medicine, University of Eastern Piedmont, 28100 Novara, Italy; 3 Department of Health Sciences, University of Eastern Piedmont, 28100 Novara, Italy; carlotta.sacerdote@cpo.it; 4 Unit of Epidemiology, Local Health Unit of Novara, 28100 Novara, Italy; 5 Department of Clinical and Biological Sciences, University of Turin, Orbassano, 10043 Turin, Italy; marco.alvich@edu.unito.it; 6 Department of Statistics, Computer Science, Applications, University of Florence, 50134 Florence, Italy; 7 Unit of Clinical Epidemiology, CPO, Città della Salute e della Scienza Hospital, 10126 Turin, Italy; gianni.ciccone@cpo.it (G.C.); eva.pagano@cpo.it (E.P.); 8 Emergency Department and High Dependency Unit, San Luigi Gonzaga University Hospital, Orbassano, 10043 Turin, Italy; adriana.boccuzzi@sanluigi.piemonte.it (A.B.); v.caramello@sanluigi.piemonte.it (V.C.)

**Keywords:** SARS-CoV-2, COVID-19, Charlson comorbidity index, multimorbidity, hospitalization, intensive care unit, mortality, national early warning score 2

## Abstract

**Background/Objectives:** Charlson Comorbidity Index (CCI) is one of the most reliable indicators to assess the impact of multimorbidity on COVID-19-related outcomes. Moreover, the patient’s clinical conditions are associated with SARS-CoV-2 outcomes. This study aimed to analyze the association between multimorbidity and COVID-19-related outcomes, evaluating whether the National Early Warning Score 2 (NEWS2) mediated these associations. **Methods:** Data were obtained through the platform “EPICLIN”. We analyzed all patients who tested positive for COVID-19 after accessing the emergency department (ED) of San Luigi Gonzaga (Orbassano) and Molinette (Turin) hospitals from 1 March to 30 June 2020. Different outcomes were assessed: non-discharge from the ED, 30-day mortality, ICU admission/death among hospitalized patients, and length of hospitalization among surviving patients. Two subgroups of patients (<65 and 65+ years old) were analyzed using logistic regressions, Cox models, and mediation analyses. **Results:** There was a greater risk of not being discharged or dying among those who were younger and with CCI ≥ 2. Moreover, the higher the CCI, the longer the length of hospitalization. Considering older subjects, a greater CCI was associated with a higher risk of death. Regarding the mediation analyses, multimorbidity significantly impacted the hospitalization length and not being discharged in the younger population. Instead, in the older population, the NEWS2 played a mediation role. **Conclusions:** This research showed that multimorbidity is a risk factor for a worse prognosis of COVID-19. Moreover, there was a strong direct effect of CCI on not being discharged, and the NEWS2 was found to act as mediator in the association between multimorbidity and COVID-19-related outcomes.

## 1. Introduction

On 11 March 2020, the World Health Organization (WHO) declared that Coronavirus Disease 2019 (COVID-19), a viral respiratory disease caused by Severe Acute Respiratory Syndrome Coronavirus 2 (SARS-CoV-2), also known as the novel coronavirus, could be characterized as a pandemic [1]. Up until the WHO declared the end of the COVID-19 pandemic on 5 May 2023, COVID-19 had already resulted in over 765 million confirmed cases and approximately 6.9 million deaths worldwide [2,3]. The pandemic lasted about 3 years.

In the literature, numerous authors have thoroughly explored the correlation between COVID-19 and multiple comorbidities. Strong associations between severe and fatal SARS-CoV-2 disease with chronic comorbidities such as kidney injury, diabetes, hypertension, cardiovascular diseases, cancer, chronic obstructive pulmonary disease (COPD), and obesity were found in studies, meta-analyses, and systematic reviews [4,5,6,7].

In the epidemiological field, comorbidity is defined in a variety of ways [8]. Beyond the presence of one or more concurrent, often chronic, diseases, there is no universally recognized definition. One useful way to establish the weight of multiple comorbidities is through the use of the Charlson Comorbidity Index (CCI) [9,10]. The CCI is an assessment tool designed specifically to predict long-term mortality. It provides a valid description of the patient’s clinical situation, and can also demarcate major diagnostic and prognostic differences among subgroups of patients sharing the same medical diagnosis [11]. The CCI has been proven useful in risk stratifications of hospitalized COVID-19 patients, as it is a reliable indicator of the clinical impact of multiple associated comorbidities. It has been observed that the per-point increase of the CCI matched an increment in mortality risk and was associated with disease severity [12,13,14]. Prognostic stratification of COVID-19 patients is not only associated with predisposing risk factors of the affected patient but also related to the severity of the disease itself, depending on the viral characteristics (e.g., strain-specific virulence) [15], the viral load (e.g., quantification of viral replication) [16], and the organ involvement (e.g., the presence of pneumonia, myocarditis, encephalitis, etc.).

In addition to these elements, given the impact of the presence of comorbidities on the patient’s clinical condition, it is important to consider the severity of the clinical conditions at the time of access at the emergency department (ED) and whether or not it may influence the effect of multimorbidity on COVID-19 outcomes [17,18]. One of the most widely utilized methods is the National Early Warning Score (NEWS) or the modified version, the National Early Warning Score 2 (NEWS2) [19,20].

The NEWS a standardized assessment of acute illness severity that was first introduced by the Royal College of Physicians (RCP) in 2012 and updated to the NEWS2 in 2017. It uses six different parameters to evaluate changes in physiological measurements: respiration rate, oxygen saturation, systolic blood pressure, pulse rate, level of consciousness, and temperature [19,20]. This is commonly adopted to stratify the risk of clinical deterioration.

The NEWS was widely used for assessing the severity and monitoring of COVID-19 patients in healthcare settings during the SARS-CoV-2 pandemic. It was applied to patient care due to its effectiveness in identifying patients at risk of deterioration, for both access to healthcare facilities [21] and hospitalization [22].

A combination of multimorbidity and severity was worth investigating, even if it may be debatable which factor impacts the patient the most.

Our study’s clinical cohort of patients admitted to the ED included the following objectives: (1) analyzing the relationship between the CCI, used as a proxy of multimorbidity, and SARS-CoV-2-related outcomes; (2) assessing the mediation role of the NEWS2 for this association and to disentangle the effect of the multimorbidity on COVID-19-related outcomes into two different effects: the direct effect of CCI on the SARS-CoV-2-related outcomes and the effect of CCI mediated by the NEWS2 on the same outcomes. Since some studies have shown that the association between clinical conditions and COVID-19 outcomes varies depending on age groups [23], the analyses were conducted in two different populations: patients < 65 years old and 65+ years old. In particular, it is expected that the NEWS2 will be more predictive in the older population and analyzing more severe outcomes, whereas the burden of morbidity will primarily impact COVID-19 outcomes in the younger population.

## 2. Materials and Methods

### 2.1. Study Population

To investigate the association between multimorbidity and COVID-19-related outcomes and to assess the mediation role of the NEWS2, data were obtained from the Epidemiologia Clinica (EPICLIN) platform. In particular, we collected data from all patients who tested positive for SARS-CoV-2 through nasal and/or pharyngeal PCR molecular swab or bronchoalveolar lavage after accessing the ED of San Luigi Gonzaga Hospital in Orbassano and Molinette Hospital in Turin (Northwest Italy) during the period from 1 March 2020 to 30 June 2020. A description of the data collection is presented elsewhere, along with information regarding informed consent [24,25,26].

### 2.2. Variables

Using ED and medical records, we collected clinical and demographic information on the study population. Specifically, in addition to gender and age, we obtained information on COVID-19 disease progression (including the presence of symptoms and whether the patients were admitted to the intensive care unit or died due to the disease), obesity, hypertension, smoking habit, NEWS2, and CCI [9], which was used as a proxy for multimorbidity.

For the purpose of the analyses, we considered four different outcomes:

1. No discharge from ED (i.e., hospitalized, transferred to other hospitals, or died) on the entire sample;
2. Death within 30 days from the first positive swab on the entire sample;
3. ICU admission and/or death among patients who were hospitalized;
4. Length of hospitalization among patients who did not die while in the hospital. This choice was made because deaths among hospitalized individuals would have greatly influenced estimates of this association. Regarding this outcome, the length of hospitalization represented the follow-up time, while the discharge from the hospital was the event that defined the subjects’ exit from the risk set.

The NEWS2 was applied to the potential mediator, as previously mentioned. Based on six physiological parameters, this score assigns each parameter a value from 0 to 3, depending on the degree of deviation from normal ranges. Additionally, for individuals requiring supplemental oxygen to maintain their recommended oxygen saturation, the overall score is increased by 2 points [19]. Consequently, the NEWS2 can range from 0 to 20. For analysis purposes, NEWS2 was categorized into three risk levels: 0 (low risk), 1 (low to moderate risk), and 2+ (moderate to high risk).

Finally, regarding the exposure variable (CCI score), this variable was categorized into 0, 1, 2–3, and 4+. However, because there were very few younger subjects with a CCI score greater than 2, the categories were adjusted to 0, 1, and 2+. However, by fitting standard models, CCI was also considered as a continuous variable in order to study the associations with the unit increase in this index

### 2.3. Statistical Analyses

After checking the normality distribution of quantitative variables through the Kolmogorov–Smirnov test [27], the sample was described using frequencies and percentages or medians and interquartile ranges (IQRs) for the qualitative and quantitative data, respectively.

We employed the Cox proportional hazards and multivariable logistic regression models, when applicable, to investigate the relationship between multimorbidity and COVID-19-related outcomes. To control for any possible confounding, the estimates were adjusted for gender, age, smoking, obesity, hypertension, and hospital. This was carried out after using the variance inflation factor (VIF) to verify the absence of multicollinearity between CCI and NEWS2.

Additionally, estimates were also adjusted for SARS-CoV-2 symptomatology as binary variables (yes vs. no) for the two outcomes on the entire population (no discharge from ED and death within 30 days of the first positive swab). Fever, cough, and dyspnea were among the symptoms taken into consideration. This was not possible for hospitalized patients, as all were symptomatic for COVID-19.

Later, to assess the mediation role played by the NEWS2 in the association between multimorbidity and COVID-19-related outcomes, mediation analyses were conducted. The mediation analysis is a statistical technique that is useful to explore the underlying mechanisms or pathways by which the exposure affects the outcome. The main objective of the mediation analysis is to determine if one or more intervening variables, referred to as mediators, fully or partially explain the impact of the exposure on the outcome.

Among the different possible approaches, we implemented the counterfactual technique of VanderWeele for the first three binary outcomes [28]. For the last outcome, instead, an innovative approach based on the counterfactual technique of VanderWeele was applied [29]. These highly advanced statistical methods allowed for us to disentangle the total effect (TE) of the multimorbidity on COVID-19-related outcomes into two different effects: the pure direct effect (PDE), which represents the effect of the exposure on the outcome that is not mediated by the mediator(s), and the total indirect effect (TIE), which expresses the effect of exposure on the same outcome that operates through the mediator(s) and quantifies the portion of the total effect that is transmitted through the mediator(s) in the causal pathway. Confidence intervals (CIs) were obtained as 95% bootstrap CIs by using the percentile method.

Before applying this technique, the assumptions underlying the method were verified. In particular, using multivariable logistic, multinomial, and Cox proportional hazards regression models, preliminary analyses were conducted to evaluate the associations between exposure–mediator and mediator–outcomes (considering the same covariates included in the models previously discussed).

Finally, receiver operating characteristic (ROC) curve, area under the curve (AUC), and related 95% Cis were estimated for the first three outcomes to evaluate the predictive ability of the CCI.

The analyses were conducted on two different subgroups: subjects aged <65 years old and 65+ years old. Moreover, before conducting this study, all observations with missing data for the variables of interest (outcomes, exposure, mediator, and confounders) were excluded from the analysis.

This study was performed using SAS (V 9.4, SAS Institute Inc., Cary, NC, USA) and R (V 4.2.1, R foundation for statistical computing, Vienna, Austria), software made available by our institutions or freely useable.

## 3. Results

Out of 844 subjects who accessed the ED of San Luigi Gonzaga and Molinette hospitals in Turin, 458 (54.2%) were <65 years old and 386 (45.8%) were ≥65 years old. Of these subjects, 564 (66.8%) were not immediately discharged from the ED, 144 (17.1%) died within 30 days of diagnosis, and 499 (59.1%) were admitted to one of the hospital’s wards, of which 222 (44.4%) died or were admitted to the ICU. In particular, 91 hospitalized patients (41.0%) died, 86 (38.7%) were admitted to the ICU, and 45 (20.3%) were admitted to the ICU and died. The clinical and demographic characteristics of the sample are shown in Table 1, Tables S1 and S2.

Regarding the association between CCI and SARS-CoV-2-related outcomes detected through classical models, the OR and HR estimates are shown in Table 2. From the results, it emerged that among those who were younger (<65 years old) and had a CCI score ≥ 2, there was a greater risk of not being discharged from the ED (OR: 3.14; 95% CI: 1.49–6.59) or dying within 30 days (OR: 4.45; 95% CI: 1.01–19.71). In addition, we found that the higher the CCI score, the longer the length of hospitalization. The results are very similar when considering CCI score as continuous variable.

When considering the subgroup of older subjects (65+ years old), estimates showed that those with a greater comorbidity burden had a higher risk of death within 30 days from the first diagnosis of COVID-19. However, this result is different when considering the CCI score as continuous, as the estimate was not statistically significant.

**Figure 1:**
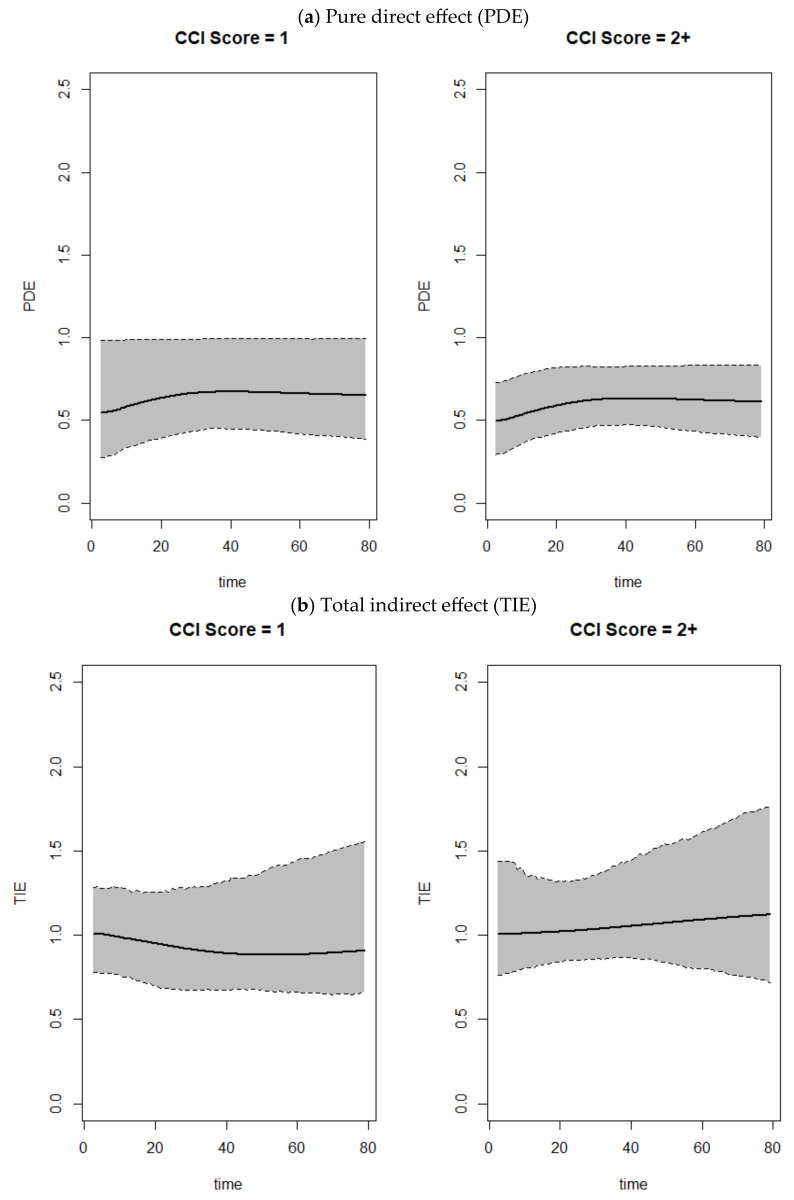

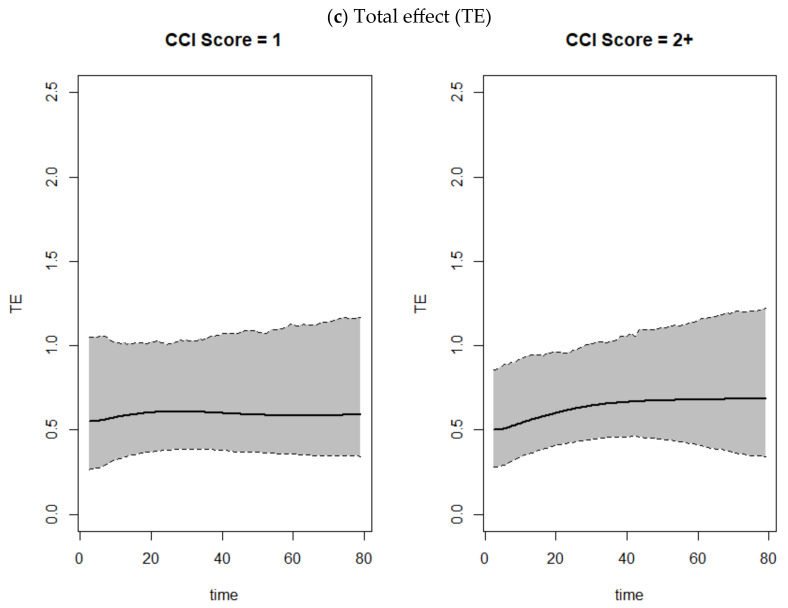
Pure direct effect, total indirect effect, and total effect over time related to the length of hospitalization for the younger population (<65 years), stratified by Charlson Comorbidity Index.

**Figure 2:**
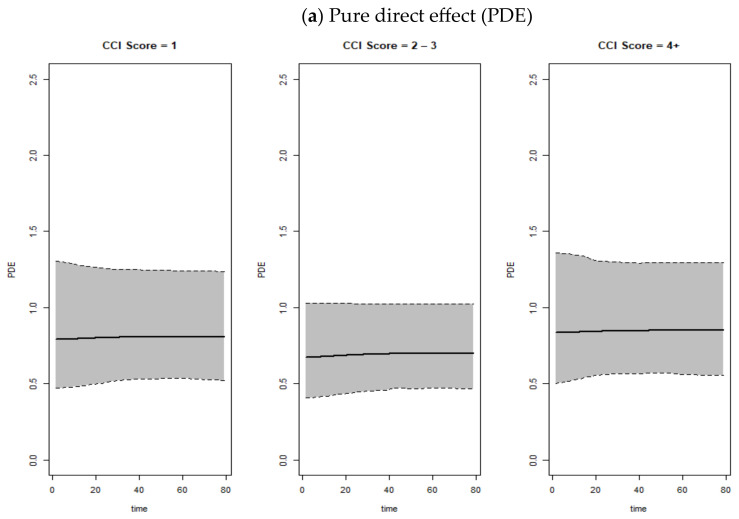

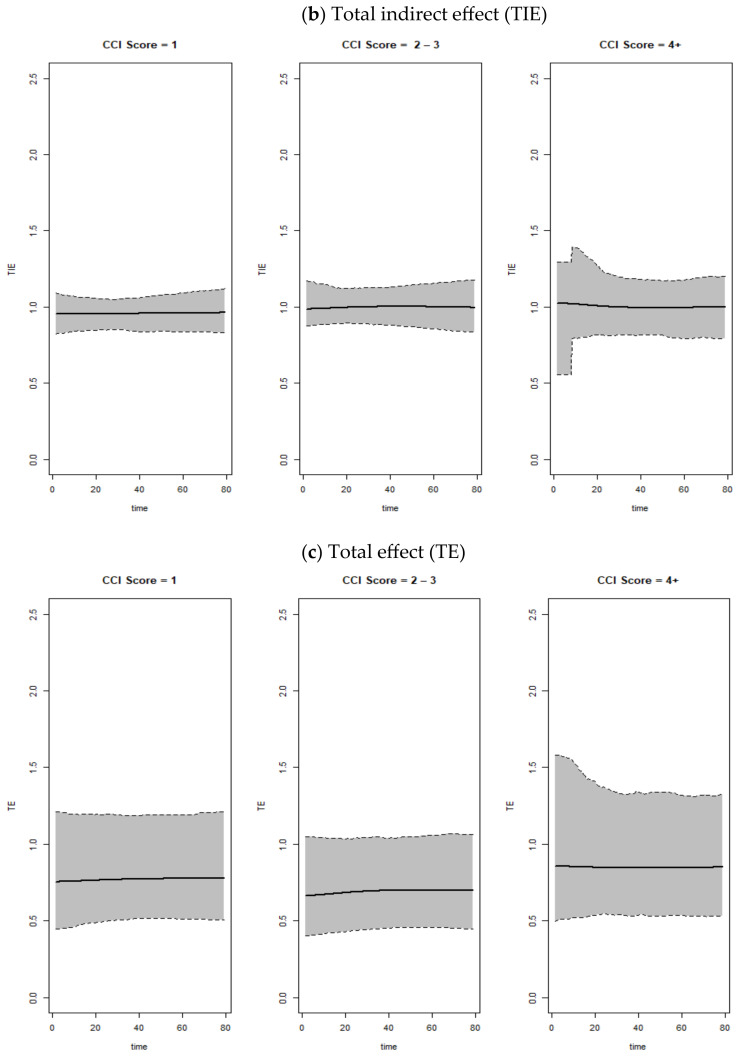
Pure direct effect (PDE), total indirect effect (TIE), and total effect (TE) over time related to the length of hospitalization for the elderly population (65+ years), stratified by Charlson Comorbidity Index.

The results related to the mediation analyses are summarized in Table 3, Figure 1 and Figure 2. The analysis of the association necessary to perform appropriate mediation analyses are presented in Supplementary Table S3. Based on the PDE estimates, we found a strong direct effect of multimorbidity on not being discharged from the ED in the younger population (CCI = 1: PDE: 1.17, 95% CI: 0.66–2.14; CCI = 2+: PDE: 2.13, 95% CI: 1.13–4.59). Therefore, there seems to be a direct association between multimorbidity and non-discharge. Furthermore, TIE estimations revealed that the NEWS played a key mediation role both in the association between CCI and not being discharged from the ED among older patients (CCI = 1: TIE: 1.27, 95% CI: 0.84–1.89; CCI = 2+: TIE: 1.51, 95% CI: 1.04–2.37) as well as in the relationship between CCI and the combined outcome “ICU admissions and/or death” (CCI = 1: TIE: 1.28, 95% CI: 1.02–1.70; CCI = 2–3: TIE: 1.27, 95% CI: 1.01–1.67; CCI = 4+: TIE: 1.15, 95% CI: 0.88–1.54). As anticipated, the total effects show that, in almost all cases, the results are very similar to those estimated through the classic methods. Hence, the choice of mediation analysis for this study is justified by the identification of a statistically significant direct and indirect effect.

Finally, the results related to the ROC Curve and AUC are shown in Supplementary Figures S1–S3. The findings indicate that, for the outcomes “no-discharge” and “death within 30 days”, the predictive capacity of the CCI was good across both subgroups. However, when considering the outcome “ICU admission/death”, the predictive capacity of CCI decreased, with the AUC just slightly above 0.50 in both groups.

## 4. Discussion

This study aimed to better acknowledge the importance of COVID-19 severity at arrival in the ED, as measured by the National Early Warning Score 2 (NEWS2), compared with the effect of multimorbidity, weighted with the CCI, in determining hospital outcomes by employing the mediation analysis. The objective of the mediation analysis was to gain a deeper understanding of the correlation between COVID-19, multimorbidity, and various outcomes. Additionally, this study aimed to determine any potential impact or influence that the NEWS2 may have on this relationship. Determining which factor has the most influence on the outcome may result in improvements in the early prediction of hospitalization necessity and resource management. Furthermore, a better stratification of the patients considering the combination of CCI and NEWS2 is useful in determining the best course of action for patient care.

Age was identified as the first prognostic determinant for COVID-19, since it has been proven that being older than 65 is a major risk factor for a more severe development of SARS-CoV-2 and worse outcome [30,31]. A similar trend was shown in our study, where patients older than 65 showed significant lower rates of ED discharge, higher mortality in the first 30 days, and more severe conditions when checked into the ED (Table 1).

It has been reported that a high CCI is associated with a higher risk of hospitalization, ICU admission, and death in both age subgroups. However, the NEWS2 has been observed as playing a key mediation role regarding the older population, strongly influencing the relationship between CCI and the outcomes of needing hospitalization and/or being admitted to the intensive care unit, whether or not a death occurs. Additionally, we found that a high CCI is associated with a higher risk of long hospitalization for the younger group of patients, but we did not find a correlation between the NEWS2 and the length of hospital stay.

In the younger subgroup, individuals with a high CCI also exhibited a higher risk of being hospitalized. This is observed as a strong direct effect, as demonstrated by the mediation test of multimorbidity on this specific outcome. It suggests that the presence of a comorbid condition may be a primary factor influencing the decision of ED physicians to admit the patient to the hospital. Taking into account the resources used during a hospital stay, this component may be rather relevant.

According to the mediation effect study, it has been observed that the NEWS2 has a stronger impact on the relationship between CCI and outcomes for the older subgroup, potentially leading to a much more accurate prediction of the patient’s progress.

The NEWS2 does not have the same weight in the younger subgroup. The younger patients’ greater homeostatic reserve may be one of the many factors contributing to this outcome.

The homeostatic reserve can be defined as the measure of the ability to function and the preservation of a specific body district, as well as the entire organism, specifically in relation to conditions that alter and lower the physiological functions. The recent literature describes how much the severe loss of reserve can impact the overall health of a person, specifically in terms of frailty, which is a multidimensional geriatric syndrome [32]. Furthermore, homeostatic reserve, which is a result of a lifetime of cumulative deterioration in several physiological systems, can also be described as a state of vulnerability to poor resolution of homeostasis after stress [33,34]. It is also described as an extreme vulnerability of the organism to endogenous and exogenous stressors, a syndrome that exposes individuals at higher risk of negative health-related outcomes as well as a transition phase between successful aging and disability [35].

Given that aging is widely recognized as one of the primary factors for the decline in organ reserve, research has shown that this reserve is sustained by excess in metabolic capacity, which, once impaired or exhausted, reduces the ability of the cell to cope with stress [36,37]. Therefore, a hypothesis can be made to explain why the course of the SARS-CoV-2 in the younger group has, in the worst cases, been found to abruptly worsen when the patient circumstances were relatively stable [38].

In this scenario, the NEWS2 has little impact on the outcome prediction in the younger group, as the physiological reserve limits the organ disfunction during the first stages of the disease, causing the NEWS2 to deteriorate only in the later phases of COVID-19 illness. In contrast, the elderly population’s low physiological reserve is reduced earlier; thus, the NEWS2 is predictive of arrival through the mediation test [39].

Aging and several related factors are the main causes of the loss of reserve that can lead to frailty syndrome. These factors alone make it more difficult for the elderly to regain stable physiological functioning and worsen their state. In particular, the development of a mild pro-inflammatory state [40,41], commonly known as inflammageing, and the dysregulation it produces in the organism [41,42] are impactful in combination with multimorbidity. This can explain how the severity of the conditions at the time of access at the ED; hence, the NEWS2 holds a key role in the association between CCI and the negative outcomes evaluated in our study. This is well-documented in the literature [43,44,45]. This important involvement of NEWS2 has also been confirmed by additional studies.

This study presents several strengths: Firstly, the analyses are based on clinical data extracted from individual medical records, ensuring high quality, reliability, and accuracy of the data investigated. Secondly, beyond achieving high estimate accuracy, the use of advanced statistical methods, particularly mediation analyses, enabled a consistent examination of the roles played by the variables under investigation.

This study, however, also has some limitations: Firstly, the data come from only two hospitals in Turin instead of from the entire Piedmont region. Therefore, the results may not be representative of the entire region. Moreover, this study focused only on the first wave of the pandemic, limiting the generalizability of the results to the entire COVID-19 pandemic, especially given the different characteristics of the subsequent waves. Another limitation is the assessment of the patient’s clinical condition upon arrival at the ED, which is based on the NEWS2 and serves as a proxy for clinical severity but may deviate from the actual patient’s clinical condition in some cases.

## 5. Conclusions

This study showed that multimorbidity is a strong risk factor for a worse prognosis of COVID-19 in both younger and older populations. Additionally, the results of the mediation analyses found that the Charlson Comorbidity Index had a strong direct effect on not being discharged from the emergency department (ED) and the length of hospitalization. Furthermore, the NEWS2, used as a proxy for the severity of the patient’s clinical condition on arrival in the ED, was found to play the role of mediator in the association between multimorbidity and COVID-19-related outcomes, especially for non-discharge and the combined outcomes of ICU admission and death. As a result, healthcare professionals must consider not only the comorbidity burden of the patients but also their clinical condition upon arrival at the ED. This consideration is crucial, as it can significantly influence the outcomes of SARS-CoV-2 infection and help determine the most appropriate path for patient care.

Considering that this study was conducted on a sample collected in 2020, future research could benefit from focusing on samples collected during more recent waves of the pandemic. Additionally, it would be important to examine the effects of vaccination on the associations studied, as the dynamics of the virus and its impact on public health have evolved significantly since the initial outbreak.

## Figures and Tables

**Table 1:** Demographic and clinical characteristics related to the entire sample, stratified by age.

Variable	Category	<65 (N = 458)	65+ (N = 386)
		N (%)	N (%)
Age	Median (IQR)	50.0 (41.0–56.8)	78.0 (73.0–85.0)
Gender	Male	258 (56.3%)	212 (54.9%)
	Female	200 (43.7%)	174 (45.1%)
Smoking	No	382 (83.4%)	311 (80.6%)
	Yes	76 (16.6%)	75 (19.4%)
Hospital	San Luigi	144 (31.4%)	135 (35.0%)
	Molinette	314 (68.6%)	251 (65.0%)
Obesity	No	429 (93.7%)	360 (93.3%)
	Yes	29 (6.3%)	26 (6.7%)
Hypertension	No	297 (64.8%)	97 (25.1%)
	Yes	161 (35.2%)	289 (74.9%)
COVID-19 Symptoms	No	16 (3.5%)	8 (2.1%)
	Yes	442 (96.5%)	378 (97.9%)
Charlson Comorbidity Index	0	353 (77.0%)	116 (30.0%)
	1	58 (12.7%)	98 (25.4%)
	2–3 *	47 (10.3%)	94 (24.4%)
	4+	-	78 (20.2%)
NEWS2	0	382 (83.4%)	173 (44.8%)
	1	29 (6.3%)	52 (13.5%)
	2+	47 (10.3%)	161 (41.7%)
Discharge	Yes	254 (55.5%)	26 (6.7%)
	No	204 (44.5%)	360 (93.3%)
Death within 30 days	No	446 (97.4%)	254 (65.8%)
	Si	12 (2.6%)	132 (34.2%)

* In the case of subjects under 65, the category refers to the value “2+”.

**Table 2:** Odds ratio (OR) and hazard ratio (HR) estimates related to each outcome, stratifying by age.

Variable	Category	No Discharge	ICU Admission or Death	Death Within 30 Days	Length of Hospitalization
		OR (95% CI)	OR (95% CI)	OR (95% CI)	HR (95% CI)
**<65**					
Charlson Comorbidity Index	0	Ref	Ref	Ref	Ref
	1	1.27 (0.66–2.43)	1.81 (0.68–4.76)	2.83 (0.58–13.77)	0.55 (0.33–0.91)
	2+	3.14 (1.49–6.59)	1.43 (0.58–3.55)	4.45 (1.01–19.71)	0.49 (0.32–0.76)
	Continuous	1.47 (1.16–1.86)	1.08 (0.87–1.34)	1.53 (1.14–2.06)	0.85 (0.74–0.99)
**65+**					
Charlson Comorbidity Index	0	Ref	Ref	Ref	Ref
	1	1.49 (0.48–4.54)	1.31 (0.70–2.45)	1.97 (1.02–3.82)	0.77 (0.52–1.15)
	2–3 *	2.52 (0.72–7.69)	1.55 (0.81–2.95)	2.14 (1.11–4.11)	0.66 (0.44–1.00)
	4+	-	1.38 (0.69–2.75)	2.16 (1.06–4.39)	0.83 (0.54–1.28)
	Continuous	1.51 (1.02–2.26)	1.05 (0.93–1.17)	1.09 (0.97–1.21)	0.98 (0.90–1.07)

* In the case of subjects under 65, the category refers to the value “2+”.

**Table 3:** Results related to mediation analyses for each outcome, stratifying by age.

Variable	Category	Effect	No Discharge	ICU Admissionor Death	Death Within 30 Days	Length of Hospitalization
			OR (95% CI)	OR (95% CI)	OR (95% CI)	HR (95% CI)
**<65**						
Charlson Comorbidity Index	0	Pure direct effect	Ref	Ref	Ref	Ref
	1		1.17 (0.66–2.14)	1.62 (0.49–4.31)	1.90 (0.01–13.12)	0.66 (0.41–0.99)
	2+		2.13 (1.13–4.59)	1.15 (0.42–2.52)	2.14 (0.22–11.05)	0.62 (0.43–0.83)
	0	Total indirect effect	Ref	Ref	Ref	Ref
	1		1.13 (0.88–1.63)	1.08 (0.58–1.77)	1.48 (0.31–3.72)	0.90 (0.67–1.32)
	2+		1.54 (0.89–3.19)	1.17 (0.63–1.13)	2.65 (0.67–7.29)	1.05 (0.82–1.45)
	0	Total effect	Ref	Ref	Ref	Ref
	1		1.32 (0.69–2.73)	1.75 (0.51–4.96)	2.80 (0.01–15.42)	0.59 (0.36–1.07)
	2+		3.27 (1.48–8.22)	1.35 (0.38–3.75)	5.67 (0.57–26.82)	0.67 (0.41–1.06)
**65+**						
Charlson Comorbidity Index	0	Pure direct effect	Ref	Ref	Ref	Ref
	1		1.23 (0.47–4.60)	1.08 (0.58–2.07)	1.48 (0.86–2.73)	0.81 (0.52–1.25)
	2–3 *		1.67 (0.56–7.94)	1.29 (0.67–2.51)	1.59 (0.90–3.00)	0.70 (0.46–1.02)
	4+		-	1.25 (0.60–2.53)	1.68 (0.94–3.17)	0.85 (0.56–1.30)
	0	Total indirect effect	Ref	Ref	Ref	Ref
	1		1.27 (0.84–1.89)	1.28 (1.02–1.70)	1.23 (0.97–1.56)	0.96 (0.84–1.07)
	2–3 *		1.51 (1.04–2.37)	1.27 (1.01–1.67)	1.17 (0.90–1.52)	1.00 (0.88–1.14)
	4+		-	1.15 (0.88–1.54)	1.15 (0.83–1.52)	1.00 (0.80–1.20)
	0	Total effect	Ref	Ref	Ref	Ref
	1		1.56 (0.56–6.36)	1.38 (0.72–2.67)	1.82 (1.06–3.39)	0.77 (0.51–1.19)
	2–3 *		2.52 (0.82–11.34)	1.64 (0.84–3.21)	1.87 (0.99–3.59)	0.70 (0.45–1.05)
	4+		-	1.43 (0.67–2.94)	1.93 (0.99–3.82)	0.85 (0.53–1.34)

* In the case of individuals under 65, the category refers to the value “2+”.

## Data Availability

Raw data cannot be openly shared due to ethical committee restrictions, which prohibit the public dissemination of individual-level data. Nonetheless, researchers can obtain aggregated data by reaching out to the corresponding author upon request.

## Supplementary Materials

The following supporting information can be downloaded at: https://www.mdpi.com/article/10.3390/jcm13237182/s1, Supplementary Table S1: Demographic and clinical characteristics related to hospitalized patients, stratified by age; Supplementary Table S2: Demographic and clinical characteristics related to hospitalized patients who did not die in the follow-up period; Supplementary Table S3: Association between mediator–exposure and outcome–mediator, conducted through multivariable logistic regression, multinomial regression, and Cox proportional hazards models; Supplementary Figure S1: ROC curve, area under the curve (AUC), and related 95% confidence interval related to Charlson Comorbidity Index (CCI) for the outcome “No discharge”, stratified by age (<65 and 65+ years old); Supplementary Figure S2: ROC curve, area under the curve (AUC), and related 95% confidence interval related to Charlson Comorbidity Index (CCI) for the outcome “30-day mortality”, stratified by age (<65 and 65+ years old); Supplementary Figure S3: ROC curve, area under the curve (AUC), and related 95% confidence interval related to Charlson Comorbidity Index (CCI) for the outcome “ICU admission/death”, stratified by age (<65 and 65+ years old).
