# Supplementary material for: Multimorbidity and COVID-19 Outcomes in the Emergency Department: Is the Association Mediated by the Severity of the Condition at Admission?
Source: J Clin Med. 2024 Nov 26;13(23):7182. doi: 10.3390/jcm13237182 (PMC11641911; doi:10.3390/jcm13237182)

Supplementary Table 1: Demographic and clinical characteristics related to hospitalized patients, stratifying by age

Variable	Category	<65<br>(*N= 180*)<br><br>N (%)	65+<br>(*N = 319*)<br><br>N (%)
Age	Median (IQR)	53.0 (47.0-58.0)	79.0 (73.0-85.0)
Gender	Male	123 (68.3%)	173 (54.2%)
	Female	57 (31.7%)	146 (45.8%)
Smoking	No	144 (80.0%)	254 (79.6%)
	Yes	36 (20.0%)	65 (20.4%)
Hospital	San Luigi	68 (36.7%)	125 (39.2%)
	Molinette	114 (63.3%)	194 (60.8%)
Obesity	No	159 (88.3%)	297 (93.1%)
	Yes	21 (11.7%)	22 (7.9%)
Hypertension	No	96 (53.3%)	74 (23.2%)
	Yes	84 (46.7%)	245 (76.8%)
Charlson Comorbidity Index	0	119 (66.1%)	87 (27.3)
	1	29 (16.1%)	89 (27.9%)
	2-3*	32 (17.8%)	76 (23.8%)
	4+	-	67 (21.0%)
News Score	0	118 (65.5%)	140 (43.9%)
	1	21 (11.7%)	46 (14.4%)
	2+	41 (22.8%)	133 (41.7%)
ICU admission or death	No	119 (66.1%)	158 (49.5%)
	Yes	61 (33.9%)	161 (50.5%)

*In the case of subjects under 65, the category refers to the value "2+".

Supplementary Table 2: Demographic and clinical characteristics related to hospitalized patients who didn't die in the follow-up period

Variable	Category	<65 (*N= 164*) N (%)	65+ (*N = 191*) N (%)
Age	Median (IQR)	53.0 (47.0-58.0)	76.0 (71.0-84.0)
Gender	Male	112 (68.3%)	101 (52.9%)
	Female	52 (31.7%)	90 (47.1%)
Smoking	No	132 (80.5%)	153 (80.1%)
	Yes	32 (19.5%)	38 (19.9%)
Hospital	San Luigi	56 (34.1%)	71 (37.2%)
	Molinette	108 (65.9%)	120 (62.8%)
Obesity	No	147 (89.6%)	176 (92.1%)
	Yes	17 (10.4%)	15 (7.9%)
Hypertension	No	91 (55.5%)	41 (21.5%)
	Yes	73 (44.5%)	150 (78.5%)
Charlson Comorbidity Index	0	113 (68.9%)	59 (30.9%)
	1	24 (14.6%)	54 (28.3%)
	2-3*	27 (16.5%)	41 (21.4%)
	4+	-	37 (19.4%)
News Score	0	115 (70.1%)	100 (52.4%)
	1	19 (11.6%)	38 (19.9%)
	2+	30 (18.3%(	53 (27.7%)
Length of hospitalization	Median (IQR)	9.0 (6.0-18.0)	16.0 (9.0-25.0)

* In the case of subjects under 65, the category refers to the value "2+".

Supplementary Table 3: Association between mediator-exposure and outcome-mediator, conducted through multivariable logistic regression, multinomial regression, and Cox Proportional Hazards Models

Outcome	Dependent variable	Independent variable	<65<br><br>OR (95% CI)	65+<br><br>OR (95% CI)
**Mediator-Exposure**				
**No discharge**	NEWS Score = 1	CCI = 1	1.43 (0.48-4.29)	0.74 (0.31-1.81)
		CCI = 2+	1.48 (0.47-4.68)	1.12 (0.53-2.36)
	NEWS Score = 2+	CCI = 1	1.29 (0.50-3.30)	1.79 (0.96-3.36)
		CCI = 2+	2.60 (1.10-6.14)	2.13 (1.21-3.76)
**ICU admission or death**	NEWS Score = 1	CCI = 1	1.58 (0.39-6.34)	0.69 (0.27-1.81)
		CCI = 2-3	1.65 (0.45-6.01)	0.83 (0.32-2.15)
		CCI = 4+	-	1.30 (0.50-3.35)
	NEWS Score = 2+	CCI = 1	1.28 (0.43-3.82)	1.79 (0.90-3.57)
		CCI = 2-3	2.09 (0.80-5.46)	1.77 (0.88-3.59)
		CCI = 4+	-	1.54 (0.72-3.31)
**Death within 30 days**	NEWS Score = 1	CCI = 1	1.43 (0.48-4.29)	0.75 (0.31-1.83)
		CCI = 2-3	1.48 (0.47-4.68)	0.98 (0.41-2.34)
		CCI = 4+	-	1.33 (0.53-3.35)
	NEWS Score = 2+	CCI = 1	1.29 (0.50-3.30)	1.79 (0.96-3.36)
		CCI = 2-3	2.60 (1.10-6.14)	2.10 (1.11-3.94)
		CCI = 4+	-	2.18 (1.08-4.41)
**Length of hospitalization**	NEWS Score = 1	CCI = 1	2.22 (0.53-9.23)	0.98 (0.34-2.82)
		CCI = 2-3	1.22 (0.29-5.06)	0.85 (0.28-2.58)
		CCI = 4+	-	1.61 (0.53-4.89)
	NEWS Score = 2+	CCI = 1	0.70 (0.17-2.85)	1.94 (0.75-5.01)
		CCI = 2-3	1.27 (0.41-4.00)	1.22 (0.44-3.39)
		CCI = 4+	-	1.57 (0.52-4.69)
**Outcome-Mediator**				
**No discharge**		NEWS Score = 0	Ref	Ref
		NEWS Score = 1	4.50 (1.70-11.87)	2.31 (0.61-8.68)
		NEWS Score = 2+	29.71 (6.94-127.30)	17.39 (2.25-134.48)
**ICU admission or death**		NEWS Score = 0	Ref	Ref
		NEWS Score = 1	1.89 (0.63-5.58)	1.00 (0.49-2.01)
		NEWS Score = 2+	4.98 (2.15-11.55)	3.88 (2.26-6.64)
**Death within 30 days**		NEWS Score = 0	Ref	Ref
		NEWS Score = 1	12.01 (0.67-216.53)	0.50 (0.20-1.25)
		NEWS Score = 2+	117.86 (13.34-1041.47)	3.90 (2.30-6.58)
**Length of hospitalization**		NEWS Score = 0	Ref	Ref
		NEWS Score = 1	0.77 (0.46-1.29)	0.96 (0.65-1.44)
		NEWS Score = 2+	0.68 (0.44-1.04)	0.82 (0.58-1.17)

Supplementary Figure 1: ROC Curve, Area Under the Curve (AUC), and related 95% Confidence Interval related to Charlson Comorbidity Index (CCI) for the outcome "No discharge", stratified by age (<65 and 65+ years old)

a) <65

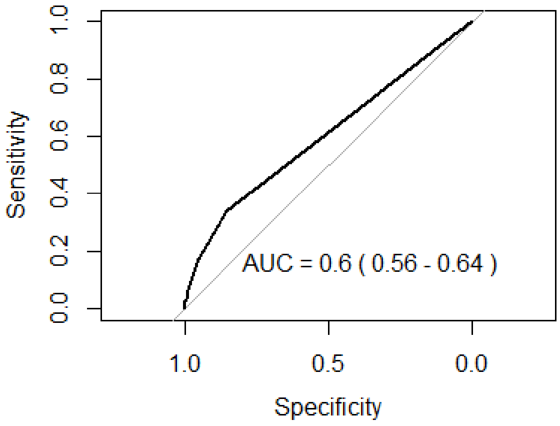

b) 65+

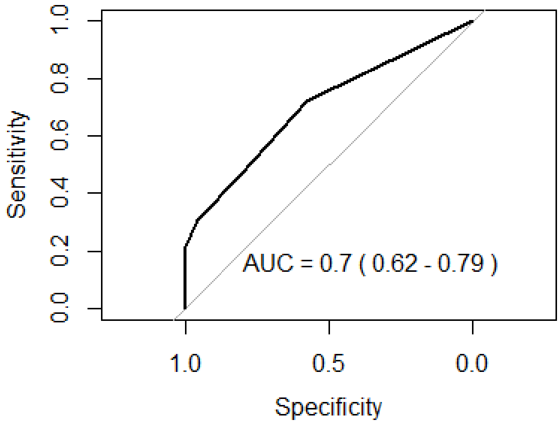

Supplementary Figure 2: ROC Curve, Area Under the Curve (AUC), and related 95% Confidence Interval related to Charlson Comorbidity Index (CCI) for the outcome "30-day mortality", stratified by age (<65 and 65+ years old)

a) <65

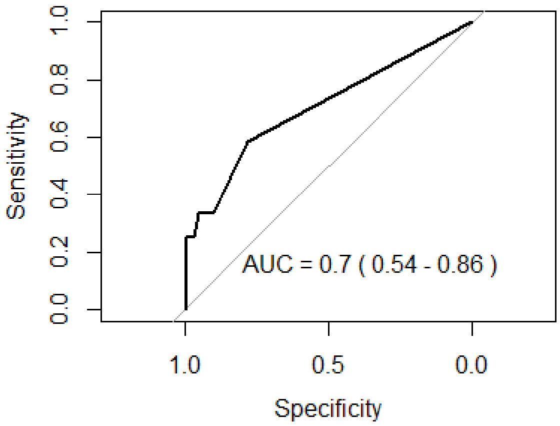

b) 65+

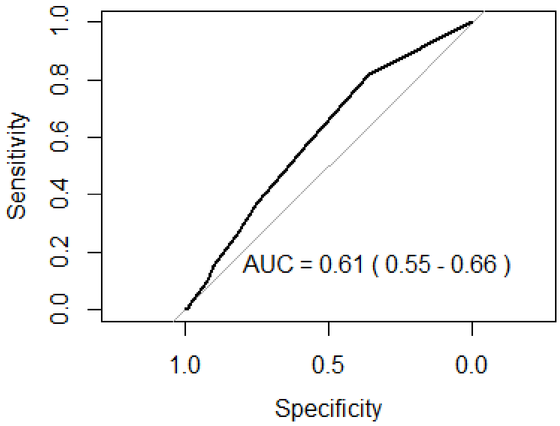

Supplementary Figure 3: ROC Curve, Area Under the Curve (AUC), and related 95% Confidence Interval related to Charlson Comorbidity Index (CCI) for the outcome “ICU admission/death” among hospitalized subjects, stratified by age (<65 and 65+ years old)

a) <65 **b) 65+**

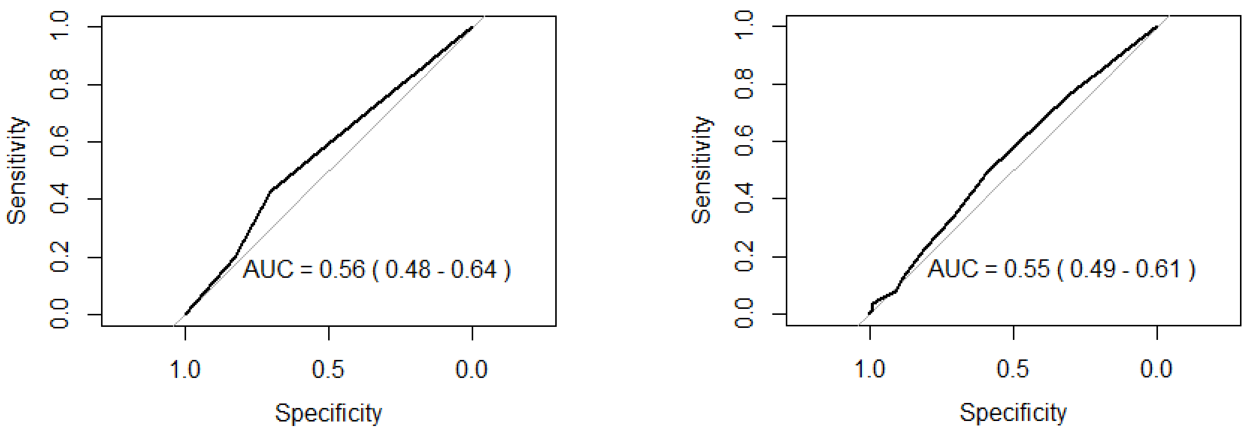

Supplement: Supplementary file 1 [file jcm-13-07182-s001.zip › jcm-3306320-supplementary.pdf]
